# Enantiomerically pure β-dipeptide derivative induces anticancer activity against human hormone-refractory prostate cancer through both PI3K/Akt-dependent and -independent pathways

**DOI:** 10.18632/oncotarget.18040

**Published:** 2017-05-20

**Authors:** Mei-Ling Chan, Chia-Chun Yu, Jui-Ling Hsu, Wohn-Jenn Leu, She-Hung Chan, Lih-Ching Hsu, Shih-Ping Liu, Polina M. Ivantcova, Özdemir Dogan, Stefan Bräse, Konstantin V. Kudryavtsev, Jih-Hwa Guh

**Affiliations:** ^1^ School of Pharmacy, College of Medicine, National Taiwan University, Taipei, Taiwan; ^2^ Graduate Institute of Clinical Medicine, College of Medicine, National Taiwan University, Taipei, Taiwan; ^3^ Department of Urology, National Taiwan University Hospital, Taipei, Taiwan; ^4^ Department of Medicinal Chemistry, Faculty of Chemistry, Lomonosov Moscow State University, Moscow, Russian Federation; ^5^ Department of Chemistry, Middle East Technical University, Ankara, Turkey; ^6^ Institute of Organic Chemistry, Karlsruhe Institute of Technology, Karlsruhe, Germany; ^7^ Institute of Toxicology and Genetics, Karlsruhe Institute of Technology, Eggenstein-Leopoldshafen, Germany; ^8^ Institute of Physiologically Active Compounds, Russian Academy of Sciences, Chernogolovka, Moscow region, Russian Federation

**Keywords:** β-dipeptide, c-Myc, PI3K/Akt, mTOR, hormone-refractory prostate cancer

## Abstract

The use of peptides that target cancer cells and induce anticancer activities through various mechanisms is developing as a potential anticancer strategy. KUD983, an enantiomerically pure β-dipeptide derivative, displays potent activity against hormone-refractory prostate cancer (HRPC) PC-3 and DU145 cells with submicromolar IC_50_. KUD983 induced G1 arrest of the cell cycle and subsequent apoptosis associated with down-regulation of several related proteins including cyclin D1, cyclin E and Cdk4, and the de-phosphorylation of RB. The levels of nuclear and total c-Myc protein, which could increase the expression of both cyclin D1 and cyclin E, were profoundly inhibited by KUD983. Furthermore, it inhibited PI3K/Akt and mTOR/p70S6K/4E-BP1 pathways, the key signaling in multiple cellular functions. The transient transfection of constitutively active myristylated Akt (myr-Akt) cDNA significantly rescued KUD983-induced caspase activation but did not blunt the inhibition of mTOR/p70S6K/4E-BP1 signaling cascade suggesting the presence of both Akt-dependent and -independent pathways. Moreover, KUD983-induced effect was enhanced with the down-regulation of anti-apoptotic Bcl-2 members (e.g., Bcl-2, and Mcl-1) and IAP family members (e.g., survivin). Notably, KUD983 induced autophagic cell death using confocal microscopic examination, tracking the level of conversion of LC3-I to LC3-II and flow cytometric detection of acidic vesicular organelles-positive cells. In conclusion, the data suggest that KUD983 is an anticancer β-dipeptide against HRPCs through the inhibition of cell proliferation and induction of apoptotic and autophagic cell death. The suppression of signaling pathways regulated by c-Myc, PI3K/Akt and mTOR/p70S6K/4E-BP1 and the collaboration with down-regulation of Mcl-1 and survivin may explain KUD983-induced anti-HRPC mechanism.

## INTRODUCTION

Carcinoma of the prostate is one of the most frequently diagnosed cancers in men. With adequate treatment, the 5-year relative survival rate of localized and regional prostate cancers is 100% in the United States; however, the rate drops to less than 30% if a distant metastasis occurs at the time of diagnosis [1]. Androgen-deprivation therapy has been the mainstay of therapy for advanced metastatic prostate cancer. However, the growth of hormone-refractory prostate cancer (HRPC) occurs ultimately after an 18- to 24-month treatment [2]. Several mechanisms are responsible to the occurrence of HRPC, including alterations in the androgen receptor gene, crosstalk between androgen receptors and growth factors, and activation of alternative signaling pathways for cell survival and proliferation [2–4]. The phosphoinositide 3-kinase (PI3K)/Akt/mTOR signaling pathway is always constitutively activated in advanced stages of prostate cancer [5, 6]. The phosphatase and tensin homolog deleted on chromosome 10 (PTEN), a negative regulator of PI3K/Akt/mTOR signaling, has been originally discovered as a tumor suppressor mutated and lost in various cancers [7] and the consequential increased PI3K activity is associated with a high Gleason score and with advanced pathological stage disease, suggesting a pivotal role of PI3K pathway in HRPC [8, 9].

The mammalian target of rapamycin (mTOR) pathway functions as a key regulator of cell metabolism, proliferation, growth and survival [10]. The key component of mTORC1 complex consists of serine/threonine kinase TOR and several other proteins, regulating its activity. The mTOR is deregulated in several diseases such as cancer and diabetes [10–12]. Decreased PTEN levels and increased PI3K activity are responsible for the deregulated mTORC1 activity. Recent studies have suggested that mTOR is important in the initiation and progression of prostate cancer, where it participates in forming precursor lesions such as high grade prostatic intraepithelial neoplasia, and proliferative inflammatory atrophy of the prostate [13]. Therefore, mTOR inhibition might be a potential therapeutic strategy against prostate cancer. However, several studies have suggested that mTOR inhibitors as a monotherapy are inadequate for the treatment of HRPC probably because of a compensatory survival mechanism after mTOR inhibition. Therefore, combinatorial targeting of PI3K, mTOR and the androgen receptor might be more feasible since a mutual interaction between PI3K and androgen receptor signaling is behind the inefficacy of mTOR inhibitors in HRPC [14].

Cyclin D1 together with cyclin dependent kinase (Cdk) 4 or 6 forms active complexes in inducing G1 to S phase progression of cell cycle through phosphorylating and inactivating the retinoblastoma (Rb) protein [15]. Cyclin D1 is important for the development and progression of a wide variety of cancers including prostate cancer [15, 16]. However, cyclin D1 protein is unstable with a short half-life less than thirty minutes. Ubiquitin-dependent degrading activity of *26S* proteasome explains the degradation of cyclin D1 protein [17, 18]*.* Numerous anticancer molecules*,* such as lovastatin, troglitazone, trichostatin A, acetylsalicylic acid and resveratrol have been demonstrated to induce cyclin D1 degradation [15]. The mTORC1 inhibitor, rapamycin, induces G1 arrest and inhibits cell proliferation partly by suppressing cyclin D1 mRNA translation and inducing its ubiquitin-dependent degradation [19, 20]. Accordingly, cyclin D1 is an attractive target for the development of anticancer therapy.

The use of peptides that directly target cancer cells and induce cytotoxicity through various mechanisms is developing as a potential anticancer strategy. Peptide-based therapy has been widely studied and utilized for the treatment of breast and prostate cancers [21]. We have developed an unprecedented synthetic method towards alternating β-proline oligomers and synthesized a series of short, well-defined β-proline peptides in both racemic and enantiomerically pure forms [22]. Subsequent testing of antiproliferative activity against HRPC cancer cell line PC-3 revealed the racemic β-dipeptide derivative with submicromolar activity [23]. Here we performed asymmetric synthesis of both enantiomeric forms, KUD983 and KUD984, of the previously identified hit racemic compound and determined the enantiomer providing major contribution to antiproliferation. After a screening test of anti-proliferative effect, KUD983 displays potent activity against HRPCs. More importantly, it is 18-fold more potent than its enantiomer (mirror isomer) KUD984. Accordingly, the anticancer mechanisms of these β-peptides have been elucidated for further development. To the best of our knowledge, this is the first paper to study the β-proline based dipeptide on inducing anticancer activity through both Akt-dependent and -independent pathways in both DU145 *(*PTEN^*+/−*^) and PC-3 *(*PTEN^*−/−*^*)* cells.

## RESULTS

### KUD983 and KUD984 induce anti-proliferative effects in HRPCs

**Figure 1:**
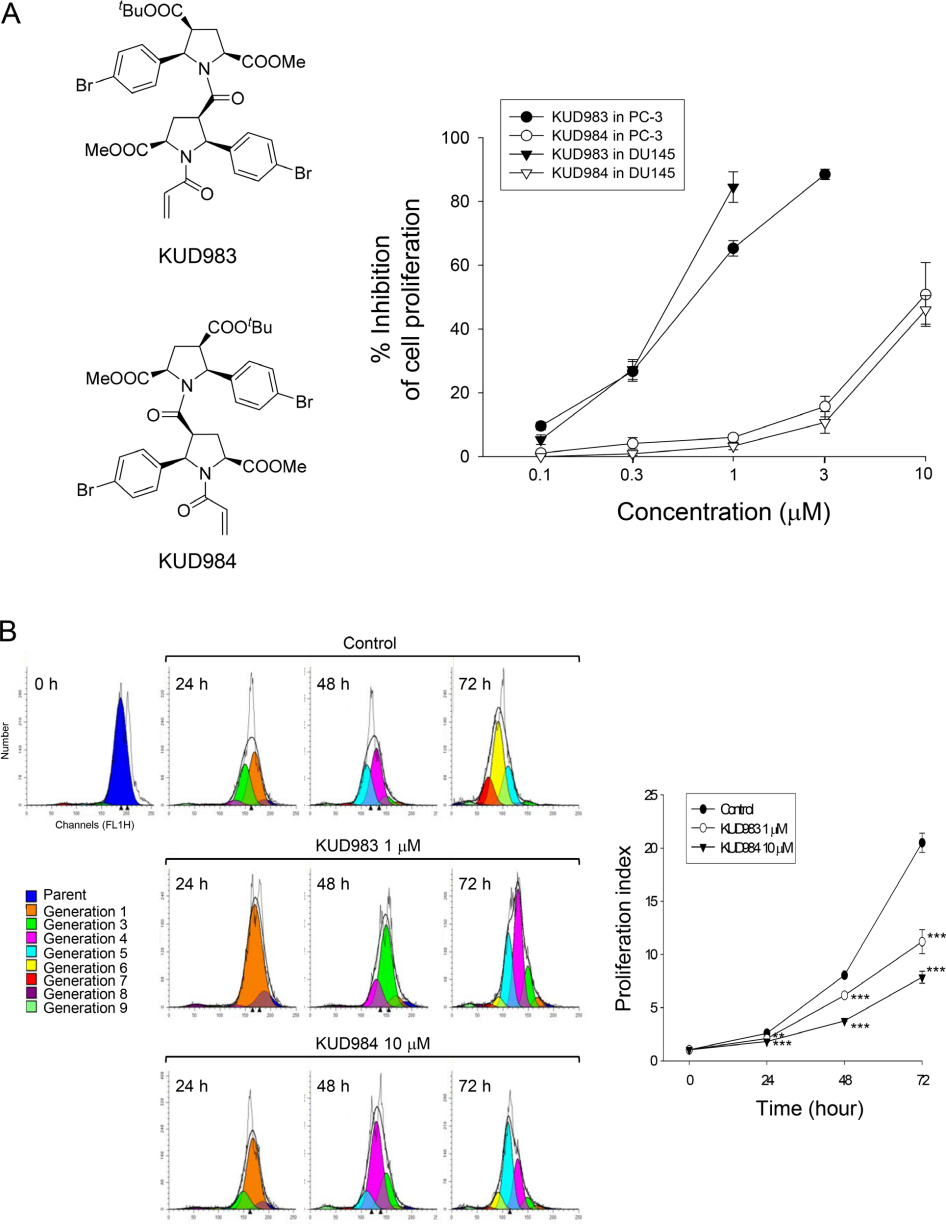
Effect of KUD983 and KUD984 on cell proliferation in PC-3 and DU145 cells The graded concentrations of the compound were added to PC-3 and DU-145 cells for 48 h (**A**) or the indicated concentration (KUD983, 1 μM; KUD984 10 μM) was added to PC-3 cells for 24 to 72 h (**B**). After the treatment, the cells were fixed and stained for SRB assay (A) or labeled with CFSE for flow cytometric analysis (B). Gray curve, total cell counts; black curve, total cells in all generations; color area, population of different generation (B). Data are expressed as mean ± SEM of three to five determinations. ^**^P < 0.01 and ^***^P < 0.001 compared with the respective control.

PC-3 and DU145 are two HRPC cell lines with different PTEN status (DU145-PTEN^+/−^; PC-3-PTEN^−/–^). Besides, both cell lines express androgen receptor [24]. Loss of PTEN expression occurs in PC-3, whereas DU145 expresses wild type PTEN. Both enantiomers KUD983 and KUD984 induced concentration-dependent anti-proliferation in PC-3 and DU145 cells using sulforhodamine B colorimetric assay. KUD983 showed 18- to 21-fold higher activity than KUD984 with IC_50_ values of 0.56 ± 0.07 *vs.* 9.95 ± 1.64 μM respectively in PC-3 and 0.50 ± 0.04 *vs.*10.67 ± 0.84 μM respectively in DU145 (Figure 1A). The inhibition of cell proliferation was further validated by CFSE-based labeling assay. CFSE fluorescent dye conjugates to cellular proteins and is allocated evenly to daughter cells after cell division. The detection and analysis of CFSE labeling can differentiate between parent and daughter cells. The analysis of fluorescence intensity and the population distribution showed that a high proportion of the lower-numbered generations retained the fluorescence after the exposure of PC-3 cells to both KUD983 (1 μM) and KUD984 (10 μM); furthermore, the proliferation indices were also significantly reduced by both compounds (Figure 1B). Similar effects were obtained in DU145 cells (Supplementary Figure 1). The data confirm the anti-proliferative activities of both KUD enantiomers.

### KUD983 induces G1 arrest of the cell cycle and subsequent apoptosis

**Figure 2:**
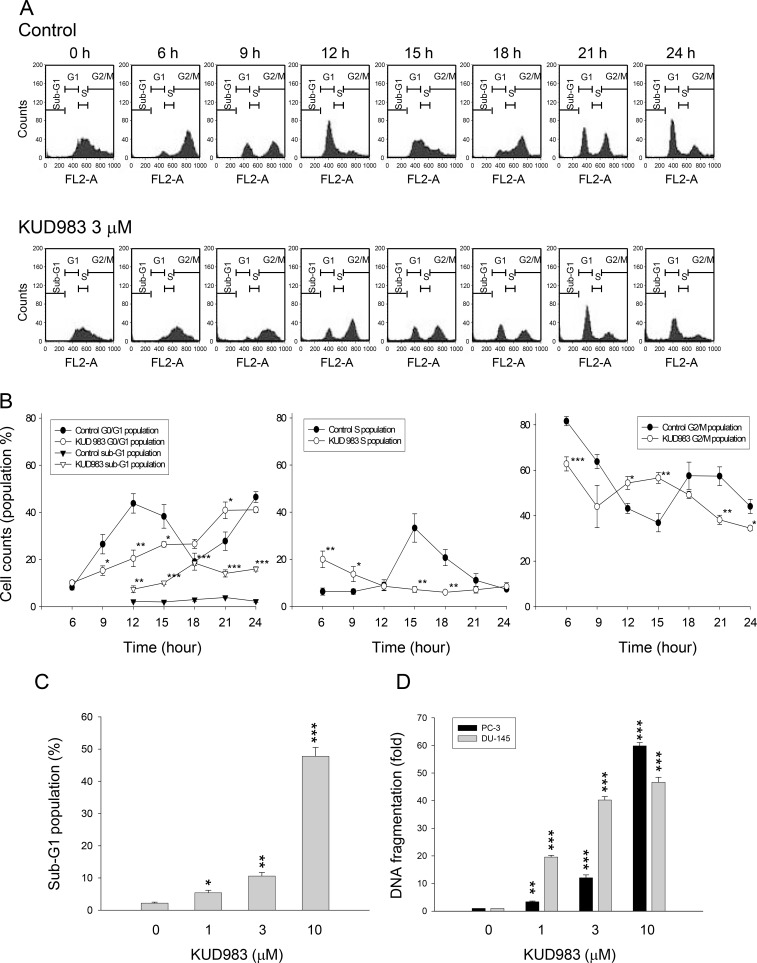
Effect of KUD983 on cell-cycle progression (**A**) Synchronization of PC-3 cells was performed by thymidine block as described in the Materials and Methods section. Then, the cells were released in the absence (upper panel) or presence of 3 µM KUD983 for the indicated times. Quantitative data of each phase (**B**) and sub-G1 population at a 12-hour KUD983 treatment (**C**) were provided. (**D**) The DNA fragmentation was determined by the detection of nucleosomal DNA. Data are expressed as mean ± SEM of three independent experiments. ^*^P < 0.05, ^**^P < 0.01 and ^***^P < 0.001 compared with the respective control.

To determine whether changes in cell cycle progression accompanied the anti-proliferative effect, PC-3 cells were synchronized by using thymidine block treatment and cell cycle profiles were compared after the release from thymidine block in the absence or presence of KUD983. Upon the release from thymidine block, the cells in control group progressed into G2/M phase and then, into G1 phase after the release for 12 h, followed by another cell cycle (Figure 2A). In contrast, KUD983 induced a gradual increase and accumulation of G1 cell proportion followed by an increase in that of sub-G1 phase (apoptosis population) (Figure 2B). Similar effects were observed in DU145 cells (Supplementary Figure 2). Furthermore, the apoptotic sub-G1 population and quantitative DNA fragmentation (apoptosis) induced by KUD983 demonstrated a concentration-dependent apoptosis (Figure 2C and 2D).

### KUD983 induces a profound inhibition of cell cycle regulators

**Figure 3:**
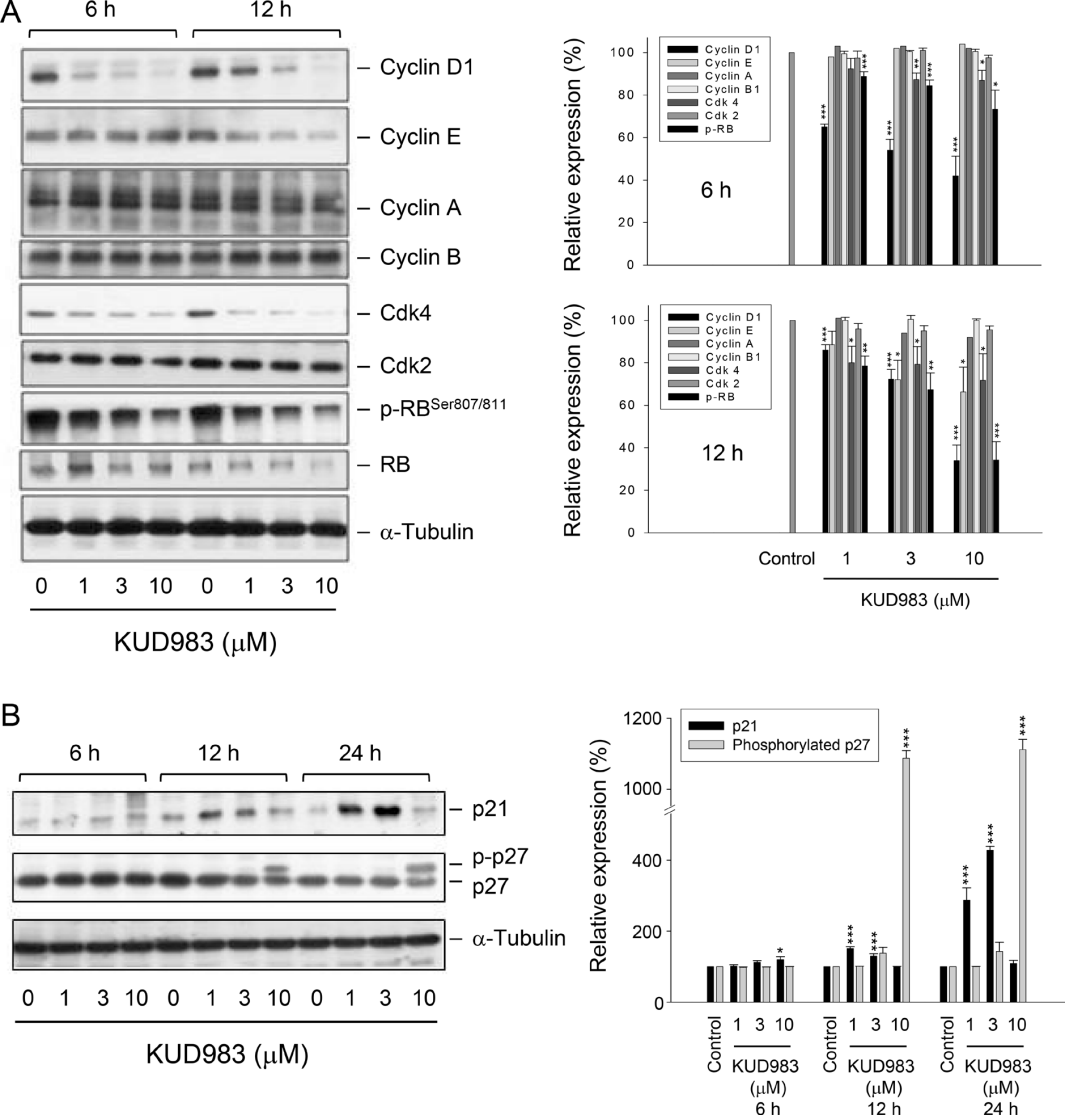
Effect of KUD983 on the expression of several cell cycle regulators PC-3 cells were incubated in the absence or presence of KUD983 for the indicated time. Cells were harvested and lysed for the detection of the indicated protein expression by Western blot analysis. The expression was quantified using the computerized image analysis system ImageQuant (Amersham Biosciences). The data are expressed as mean ± SEM of three to five independent experiments. ^*^P < 0.05, ^**^P < 0.01 and ^***^P < 0.001 compared with the control.

Cdk activity is regulated by the levels of cyclin partners and by the association with intrinsic Cdk inhibitors. The cyclin D1/Cdk4 is a key complex in the determination of cell cycle progression through G1 phase [15]. Cyclin E binds to Cdk2, forming a complex and playing a critical role in the G1 phase and G1-S phase transition. Both cyclin D1/Cdk4 and cyclin E/Cdk2 complexes phosphorylate Rb protein to promote G1 progression [15, 25]. After exposure of cells to KUD983 for 6 and 12 h, the protein levels of several cell cycle regulators were dramatically decreased in PC-3 (Figure 3A) and DU-145 cells (Supplementary Figure 3). In contrast, the protein expression of cyclin E was down-regulated at a 12-h treatment with KUD983 (Figure 3A and Supplementary Figure 3). The Cdk inhibitors p21 and p27 bind to cyclin/Cdk complexes to inhibit their catalytic activity and induce cell-cycle arrest in response to various stimuli. They are clearly positioned to serve as both a sensor and an effector of numerous anti-proliferative signals [26]. The lower concentrations of KUD983 (e.g., 1 and 3 μM) induced an increase of p21 protein level in PC-3 that was correlated to down-regulated expressions of cyclin D1, Cdk4 and phosphorylated Rb, indicating the well documented role of p21 as a Cdk inhibitor (Figure 3B). Notably, the higher concentrations of KUD983 (e.g., 10 μM) which induced cell apoptosis had no effect on p21 expression in PC-3, but instead led to a dramatic increase of phosphorylated p27 expression (Figure 3B). Various mechanisms have been reported to be responsible for increasing p27 phosphorylation in which the autophagy decision making is an important involved process (please see below for details). In contrast, p27 was a dominant Cdk inhibitor in DU145 cells and was up-regulated by KUD983 (Supplementary Figure 4).

### KUD983 inhibits PI3K/Akt and mTOR pathways and down-regulates several anti-apoptotic proteins

**Figure 4:**
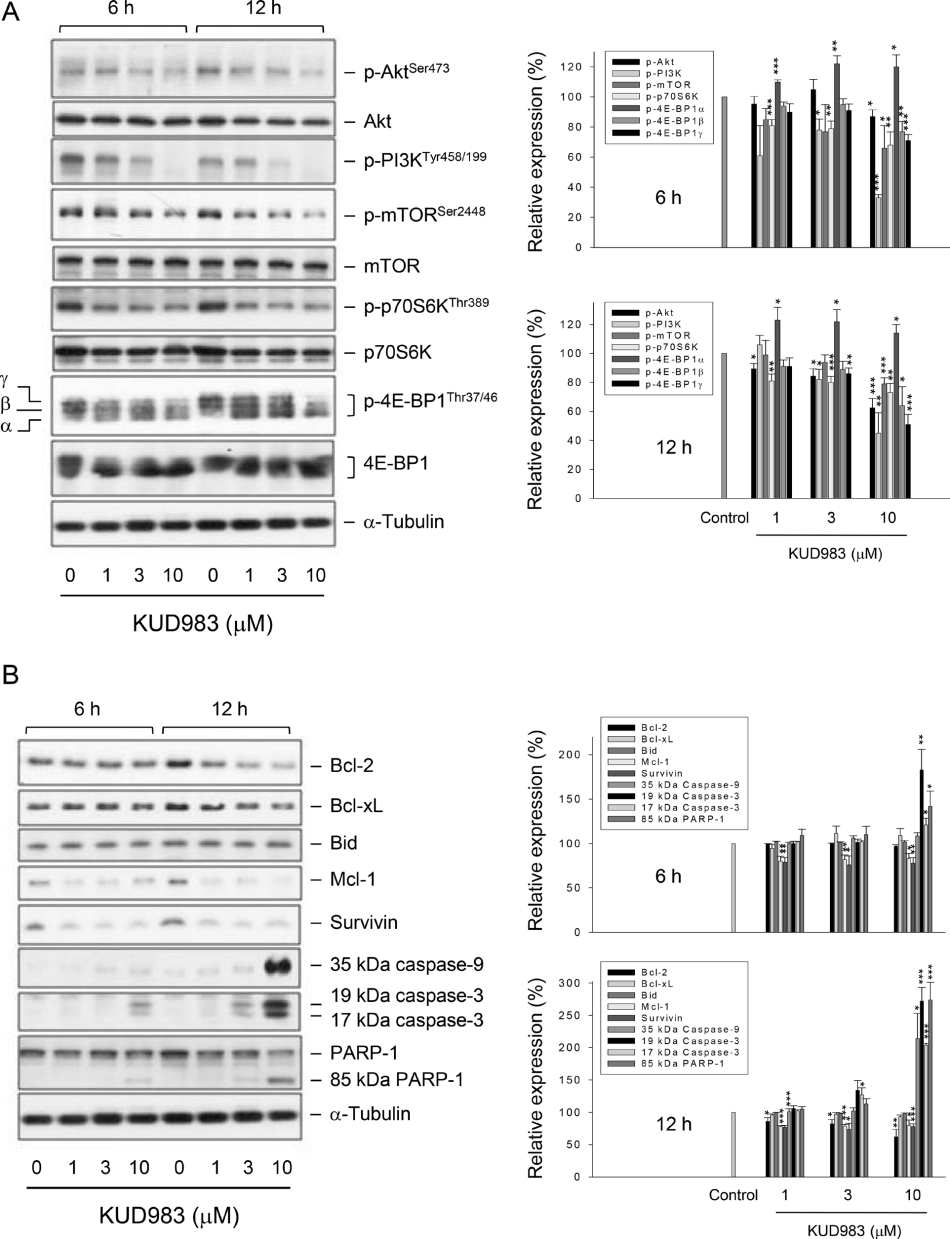
Effect of KUD983 on the expression of several proteins PC-3 cells were incubated in the absence or presence of KUD983 for the indicated time. Cells were harvested and lysed for the detection of the indicated protein expression by Western blot analysis. The expression was quantified using the computerized image analysis system ImageQuant (Amersham Biosciences). The data are expressed as mean ± SEM of three to four independent experiments. ^*^P < 0.05, ^**^P < 0.01 and ^***^P < 0.001 compared with the control.

PI3K is a lipid kinase and generates phosphatidyl inositol-3,4,5-trisphosphate, a second messenger for Akt translocation and activation. PI3K/Akt pathway plays a key role in multiple cellular functions such as cell proliferation and survival, and is always constitutively activated in advanced stages of prostate cancer [5, 6]. mTOR/p70S6K pathway integrates both intracellular and extracellular signals, serving as a key regulator of cell metabolism, proliferation, growth and survival [10]. KUD983 resulted in a decreased phosphorylation of PI3K (Tyr458/199), Akt (Ser473), mTOR (Ser2448) and p70S6K (Thr389), indicating the inhibition of these kinases activities (Figure 4A). Notably, 4E-BP1 separated three different forms: α, β, and γ. The β and γ bands represented highly phosphorylated forms of the protein whereas the α band corresponded to the weakly phosphorylated form. The data showed that KUD983 induced a shift in the distribution of the protein in favor of the α form (Figure 4A) that was similar to rapamycin (an mTOR inhibitor) with almost all of the protein appearing as the α-form [27, 28].

Bcl-2 family of both pro-apoptotic (e.g., Bax, Bid and Bad) and anti-apoptotic members (e.g., Bcl-2, Bcl-xL and Mcl-1) govern mitochondrial outer membrane permeabilization. Overexpression of anti-apoptotic Bcl-2 family members explains the chemo-resistance mechanism in numerous types of cancers during chemotherapy [29]. Survivin, a member of the inhibitor of apoptosis (IAP) family, is another chemo-resistant protein and serves as an inhibitor to block caspase activation and apoptosis. The survivin protein also is highly expressed in a wide variety of human tumors [30]. These studies encourage targeted approaches on anti-apoptotic proteins to circumvent the clinical problem. As a result, KUD983 induced a dramatic decrease of the expression of several anti-apoptotic proteins, including Bcl-2, Mcl-1 and survivin, leading to activation of caspases (Figure 4B). Moreover, down-regulation of both Mcl-1 and survivin during KUD983 treatment was with similar efficiency to that of cell cycle regulators (Figure 3A) but was much more susceptible than caspase activation (Figure 4B), suggesting that Mcl-1 and survivin might be crucial regulators on the control of cell cycle progression in spite of their anti-apoptotic properties. Furthermore, because Bcl-2 family members govern mitochondrial outer membrane permeabilization, KUD983 induced a time- and concentration-dependent loss of mitochondrial membrane potential confirming the crucial role of Bcl-2 family of proteins (Supplementary Figure 5).

### Akt contributes to KUD983-induced caspase activation but not inhibition of mTOR and cell cycle regulators

**Figure 5:**
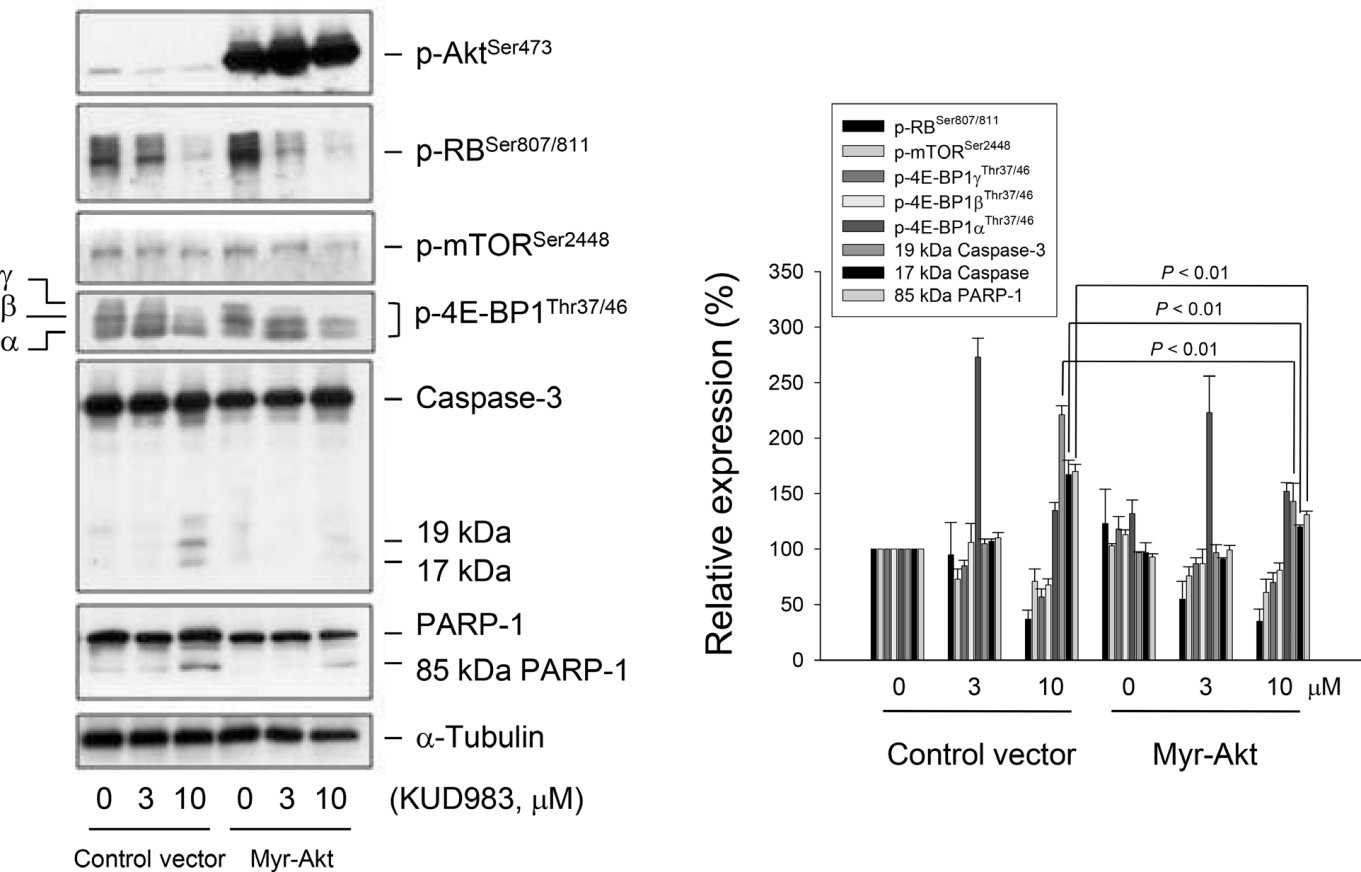
Determination of the functional involvement of Akt PC-3 cells were transfected with control vector or myr-Akt. Then, the cells were incubated in the absence or presence of KUD983 (3 or 10 μM) for 24 h. After the treatment, the cells were harvested and lysed for the detection of the indicated protein expressions by Western blot analysis. The expression was quantified using the computerized image analysis system ImageQuant (Amersham Biosciences). Data are expressed as mean ± SEM of three independent experiments.

Akt regulates a number of downstream signaling proteins to control cell proliferation, survival, cell death and motility [5, 31, 32]. The mTOR is one of the most widely studied downstream effectors that in turn affect the transcription and activity of p70S6K [10–13]. However, increasing lines of evidence support the participation of Akt-independent mTOR signaling in the regulation of various cellular functions such as cell adhesion, invasion, cell cycle progression and apoptosis [33–35]. To determine the functional role of Akt, PC-3 cells were transfected with constitutively active Akt (myr-Akt) and several protein expressions were examined. Consequently, overexpression of myr-Akt significantly inhibited KUD983-induced caspase activation but not the other cellular signaling pathways, such as cell cycle regulation (e.g., RB phosphorylation) and mTOR translational pathway (e.g., mTOR and 4E-BP1) (Figure 5), indicating the involvement of Akt-independent mTOR signaling to KUD983 action.

### KUD983 down-regulates nuclear and total levels of c-Myc protein expression

**Figure 6:**
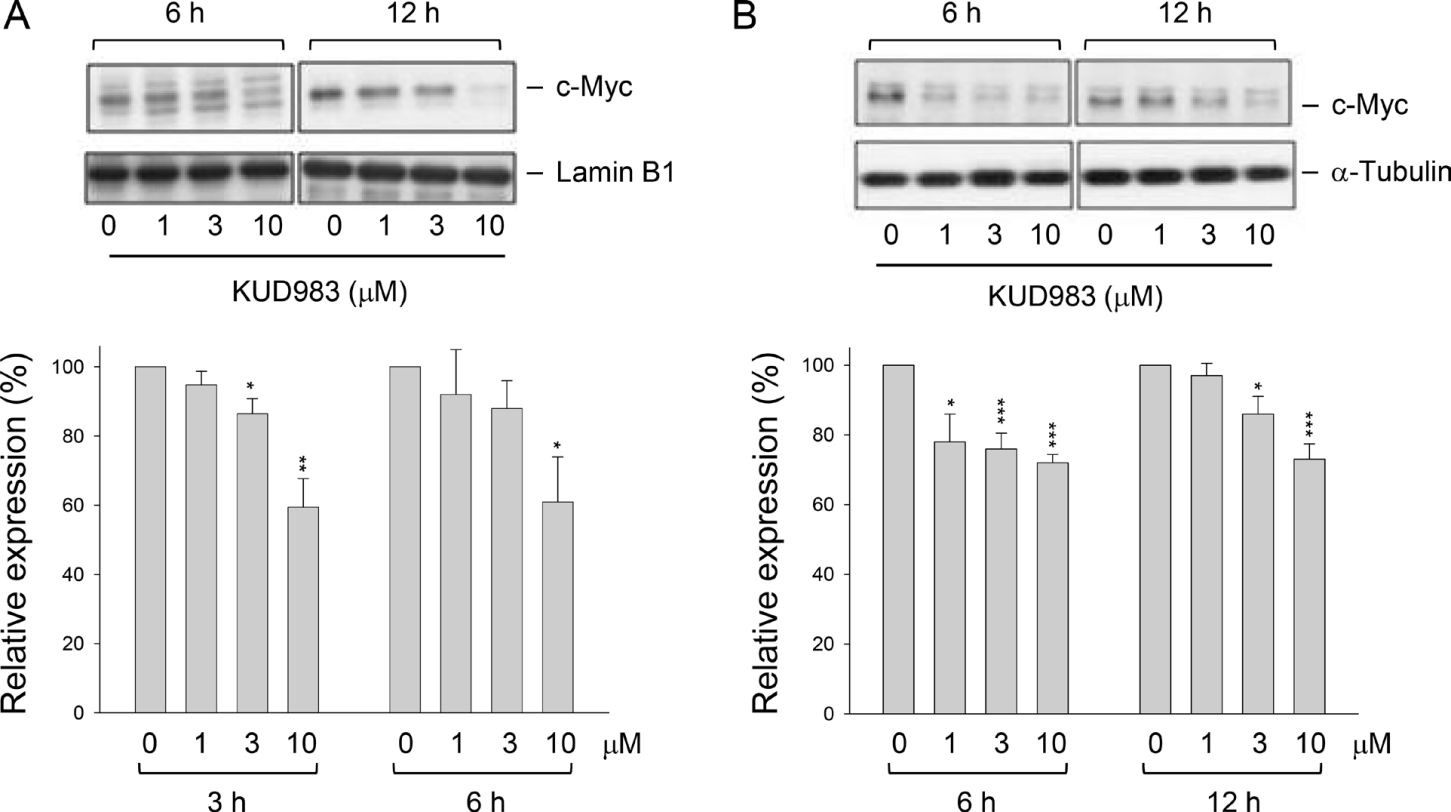
Effect of KUD983 on total and nuclear c-Myc protein expression PC-3 cells were incubated in the absence or presence of KUD983 for the indicated time. The cells were harvested for the separation of the nuclear fraction (**A**) or the cells were lysed (**B**) for the detection of c-Myc protein expression by Western blot analysis. The expression was quantified using the computerized image analysis system ImageQuant (Amersham Biosciences). The data are expressed as mean ± SEM of three independent experiments. ^*^P < 0.05, ^**^P < 0.01 and ^***^P < 0.001 compared with the control.

C-myc is an oncogene which functions both in the stimulation of cell proliferation and apoptosis. c-Myc collaborating with various growth factor receptors on the coordination of cyclin D1 expression *is* a key participant in cell cycle progression, in which aberrancies have been associated with malignant transformation [36]. Recently, several lines of evidence suggest that c-Myc increases protein synthesis during tumorigenesis not only through transcriptional contribution but also by stimulating mTOR-dependent 4E-BP1 phosphorylation pathway [37]. The data showed that KUD983 induced a dramatic down-regulation of cyclin D1 in both protein (Figure 3A) and mRNA levels (Supplementary Figure 6), and the inhibition of mTOR signaling pathway (Figure 4A). Therefore, the detection of c-Myc protein levels under KUD983 treatment was performed to gain a clearer understanding of its role. As a result, KUD983 significantly decreased both nuclear and total c-Myc protein expression in PC-3 cells (Figure 6A and 6B) indicating the link in coordinating KUD983-induced effects.

### KUD983 induces autophagic cell death

**Figure 7:**
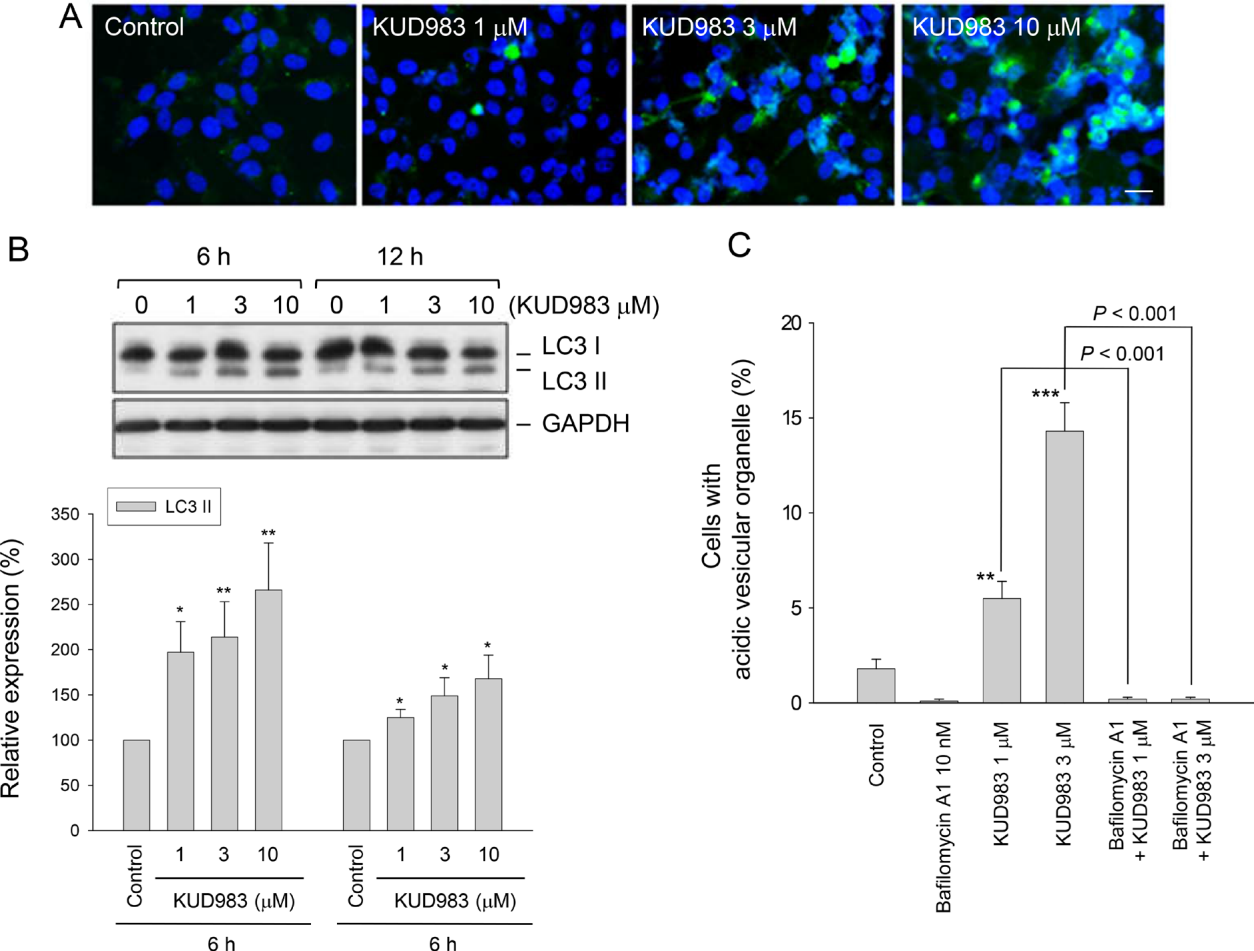
Effect of KUD983 on autophagic cell death PC-3 cells were incubated in the absence or presence of KUD983 or the indicated compound for 24 h (**A** and **C**) or for 6 and 12 h (**B**). After the treatment, the autophagic cell death was detected through staining with GFP (green fluorescent protein)-LC3. Nuclear identification was performed by DAPI staining. The cells were analyzed by a confocal laser microscopic system. *Bar*, 20 μl (A). The cells were harvested and lysed for the detection of the indicated protein expression by Western blot analysis. The expression was quantified using the computerized image analysis system ImageQuant. The LC3II expression relative to GAPDH was demonstrated (B). The cells were harvested and the autophagy was assessed by detecting acidic vesicular organelles (AVO) using flow cytometric analysis of acridine orange staining (C). The data are expressed as mean ± SEM of three to four independent experiments. ^*^P < 0.05, ^**^P < 0.01 and ^***^P < 0.001 compared with the control.

**Figure 8:**
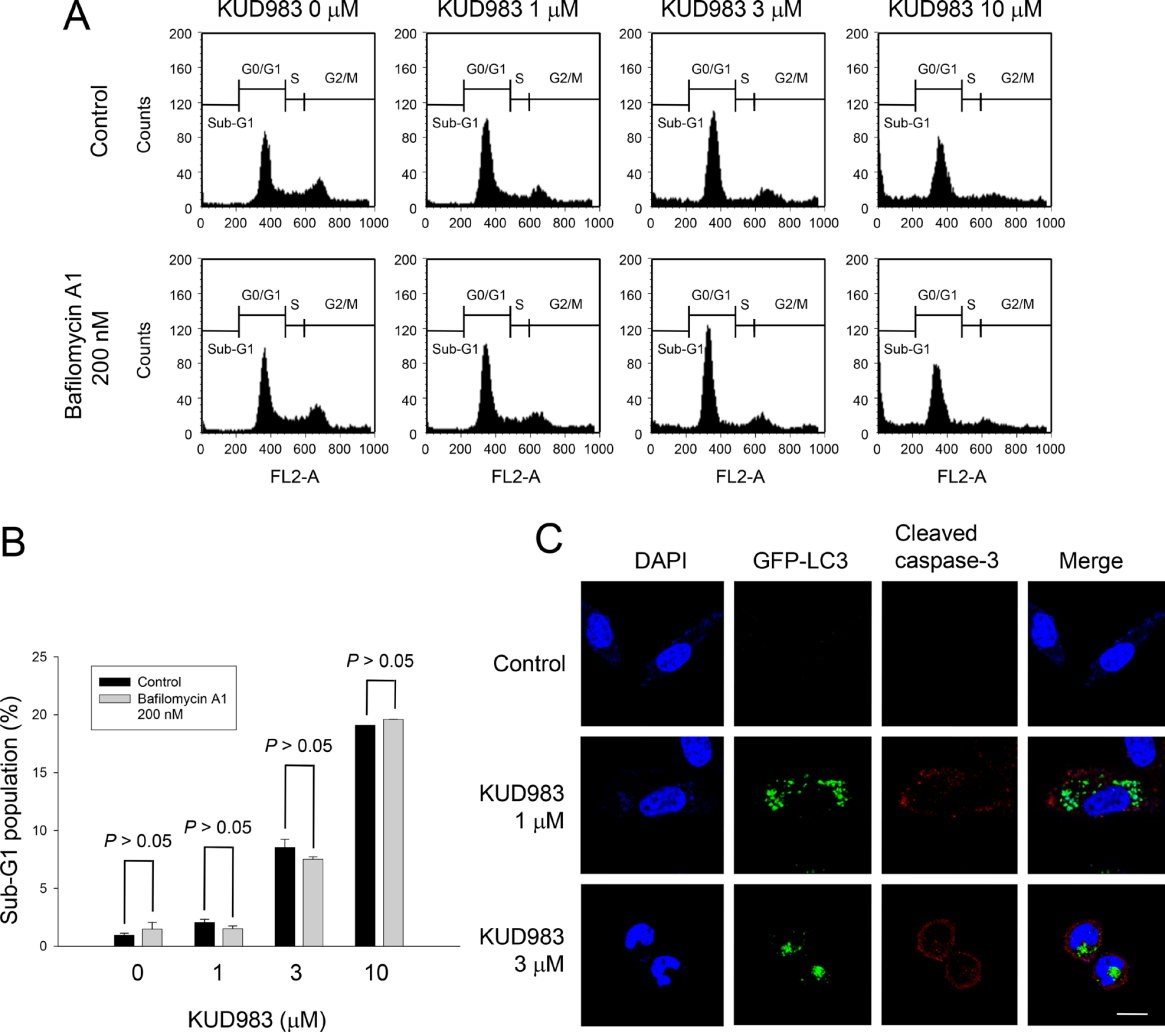
Effect of KUD983 on cell apoptosis and autophagy in PC-3 cells Cells were incubated in the absence or presence of KUD983 and/or bafilomycin A1 for 24 h. After the treatment, the cells were harvested for flow cytometric analysis of PI staining (**A**, **B**), or the cells were fixed for the detection of apoptosis (cleaved caspase-3) and autophagy (**C**). Data are expressed as mean ± SEM of three determinations. *Bar*, 20 μm.

Autophagy, an evolutionary conserved process to recycle intracellular substances in keeping homeostasis in different cellular contexts, avoids accumulation of damaged proteins and organelles. Current evidence supports the notion that stimulation of autophagic cell death can impair tumorigenesis [38]. Moreover, mTOR has been well recognized to play a key role in linking the pathways to coordinate and regulate the balance between cell growth, apoptosis and autophagy under the exposure to a number of cellular stimulation and stress [39]. In light of KUD983-induced inhibition of mTOR pathway, its effect on autophagy was determined. As demonstrated in Figure 7, KUD983 induced a concentration-dependent increase of autophagic cell death using confocal microscopic examination (Figure 7A), tracking the level of conversion of LC3-I to LC3-II (Figure 7B) and detecting acidic vesicular organelle (AVO)-positive cells by flow cytometry (Figure 7C). Furthermore, the data demonstrated that bafilomycin A1, an autophagy inhibitor by preventing AVO formation, did not affect KUD983-induced apoptosis suggesting that the apoptosis and autophagy might be two independent but co-existing events (Figure 8). The data also suggest the contribution of autophagic cell death to KUD983-induced effect.

## DISCUSSION

In contrast to proteins and monoclonal antibodies which have major limitations of poor delivery to tumors and dose-limiting toxicity to the liver and bone marrow, discovery of peptide drugs is a potential anticancer approach due to several advantages, such as small size, ease of synthesis and modification, tumor penetrating ability, and good biocompatibility [21, 40, 41]. KUD983 is a novel enantiomerically pure β-diproline derivative with alternating symmetry of monomeric pyrrolidine units [22]. It induced G1 arrest of cell cycle and subsequent apoptosis. Cyclin D1 and its binding partner Cdk4, the protein complex required for progression through the G1 phase, were the most susceptible to KUD983-induced protein down-regulation. Both cyclin D1 and Cdk4 proteins have been shown to be unstable with short half-life (about 30 min and 3 h, respectively). The rapid decline of these proteins was reasonable, indicative of inhibition of translational and/or transcriptional levels by KUD983. Several lines of evidence suggest that p21 plays a key role in the regulation of cyclin E/Cdk2, the complex essential for the G1-to-S phase transition. p21 shows a high affinity for cyclin E/Cdk2 complex [42] and is responsible for cyclin E down-regulation [43]. Similar to these reports, KUD983-induced p21 activation was correlated to and responsible for the cyclin E down-regulation. Notably, the data demonstrated that p27 was highly expressed on PC-3 cells and an increased phosphorylated form was observed under KUD983 treatment. p27 is a member of the universal cyclin dependent kinase inhibitor family, inversely regulating the cell cycle progression. In addition, p27 is a putative tumor suppressor gene and promoter of apoptosis [44]. In addition to the role as a Cdk inhibitor, p27 also has emerged as an intrinsically multifunctional protein with non-canonical and Cdk-independent functions. Certain non-canonical functions such as oncogenic activation of signaling pathways may contribute to tumor-aggressiveness and metastasis [45]. The data in the present study favored the non-canonical and Cdk-independent function of p27. In addition, various kinases have been reported to be responsible for p27 phosphorylation, including Akt, AMP-activated protein kinase, ATM and Mirk [46, 47]. Besides, the phosphorylation of p27 is known to occur in autophagic cell death [48]. KUD983 was capable of inducing autophagic cell death, indicating the possibility of autophagy-dependent p27 phosphorylation. However, the responsible kinases need further examination.

The PI3K/Akt/mTOR pathway is a fundamental survival signaling constitutively activated in many types of cancer. Genes in this pathway are the most frequently altered in human cancers. Aberrant activation of this pathway including loss of PTEN function, amplification or mutation of PI3K or Akt and overexpression of growth factor receptors is associated with tumorigenesis, cancer progression, and drug resistance [49]. This pathway is, therefore, an attractive anticancer target for therapy. In addition to PI3K/Akt-dependent pathway, it is noteworthy that increasing lines of evidence support the involvement of PI3K/Akt-independent mTOR signaling in the regulation of cell adhesion, invasion, cell cycle progression and apoptosis [33–35]. Zhang and the colleagues have used PTEN-deficient models of prostate cancer, reporting that the inhibition of either Akt or mTOR kinase alone has no effect on the status of the other kinase [50]. The data strongly suggest that the mTOR-mediated network in PTEN-null tumor is independent of AKT activity. Our data were consistent with this report, showing that KUD983 induced a suppressive effect on Akt-independent mTOR signaling in PTEN-deficient PC-3 cells. However, the inhibition of Akt activity still partly contributed to the caspase-dependent apoptosis to KUD983 action.

Pro-apoptotic and anti-apoptotic Bcl-2 family of proteins interact with each other in keeping mitochondrial integrity and in determining cell fate. Among the anti-apoptotic Bcl-2 family members, Mcl-1 has a short half-life and is uniquely regulated by numerous oncogenic signaling pathways including mitogen activated protein kinase pathway, mTOR pathway and PI3K/Akt pathway that rapidly up-regulate Mcl-1 for cell survival [51]. Numerous studies have reported that Mcl-1 protein levels are inhibited by targeting PI3K/Akt and/or mTOR pathways using specific inhibitors, leading to increased tumor cell killing in both *in vitro* and *in vivo* models [51, 52]. Furthermore, it has been suggested that up-regulation of Mcl-1 is responsible for the resistance in PTEN deficient cells in response to various cellular stresses [53]. Taken together, it has been suggested that Mcl-1 plays a key role in promoting resistance and cell survival during the activation of PI3K/Akt and mTOR pathways. It also supports that the suppression of Mcl-1 expression is highly susceptible to KUD983-induced inhibition of both PI3K/Akt and mTOR pathways.

Survivin belongs to the inhibitor of apoptosis (IAP) family, a group of anti-apoptotic proteins containing one or more characteristic baculovirus IAP repeats (BIR) domains. Similar to the other members, survivin exhibits multiple biological activities including binding and inhibiting caspases, regulating cell cycle progression, and involving in resistance mechanism [54]. Wheatley has reported two separate functions of the survivin that the C-terminus is essential to cell division and the N-terminus is required for apoptosis [55]. The deficiency of survivin tends to halt cell cycle progression at G1 phase and to promote caspase activation and apoptosis [54, 55]. Accordingly, KUD983-induced survivin down-regulation might be attributed to G1 arrest of the cell cycle and caspase-dependent apoptosis. Furthermore, Xu and the colleagues have reported that the loss of PTEN in the prostate results in a substantial up-regulation of survivin expression that contributes to tumor development [56], suggesting that PI3K/Akt activity is critical to survivin expression. This study also support that the inhibition of PI3K/Akt induced by KUD983 may contribute, at least partly, to the survivin down-regulation. Of note, it has been demonstrated that survivin suppressants induce both apoptosis and autophagy, which can be rescued by ectopic expression of survivin [57]. This study supports and suggests that survivin down-regulation may be possibly responsible for KUD983-induced autophagic cell death. However, the inhibition of mTOR activity may contribute largely to the induction of autophagy by KUD983 since it has been the most extensively studied and well proven that mTOR kinase is a critical regulator of autophagy induction, with activated mTOR inhibiting autophagy. A molecular mechanism that connects mTOR and autophagy pathways has been revealed, in which the decreased MTORC1 activity leads to modified phosphorylation on uncoordinated 51-like kinase 1 (ULK1) and downstream substrates including members of the Beclin1 complex to drive membrane trafficking and autophagosome assembly [58]. Therefore, mTOR suppression has been considered to be the inevitable mechanism for autophagy induction.

Many studies have well recognized that c-myc has a potent oncogenicity, which can be further improved by collaborations with other anti-apoptotic proteins and oncogenes. Many of these collaborations may converge at the cyclin D1/CDK4 complex [36, 59]. Berns and the colleagues have made a mutant c-Myc protein (MadMyc) to actively repress c-myc target genes and the data show that expression of MadMyc in cycling NIH3T3 cells causes a significant accumulation of cells in G1; however, ectopic expression of cyclin E/Cdk2 and cyclin D1/Cdk4 can rescue the progression of the cell cycle [60]. The data suggest that c-Myc is crucial in the regulation of both cyclin E/Cdk2 and cyclin D1/Cdk4 complexes and facilitates G1 exit. Our data showed that KUD983 caused a profound decrease of both total and nuclear c-Myc protein expression and down-regulation of both cyclin D1 and cyclin E in mRNA and protein levels, suggesting that c-Myc might be a critical target to KUD983-induced suppression of cell proliferation. In conclusion, the data suggest that KUD983 is an effective anticancer dipeptide derivative against HRPCs through the inhibition of cell proliferation and induction of apoptotic and autophagic cell death. KUD983 induced G1 arrest of the cell cycle associated with the up-regulation of p21 and down-regulation of both cyclin D1 and cyclin E and de-phosphorylation of Rb, in which c-Myc suppression may play a key role. In addition, the inhibition of PI3K/Akt and mTOR/p70S6K/4E-BP1 pathways by collaborations with down-regulation of anti-apoptotic proteins (e.g., Bcl-2, Mcl-2 and survivin) are responsible to both apoptotic and autophagic cell death to KUD983 action. These data also reveal for the first time that the dipeptide enantiomer displays anticancer activity through multiple pathways to inhibit cell proliferation and to induce both apoptotic and autophagic cell death.

## MATERIALS AND METHODS

### Materials

RPMI 1640 medium and fetal bovine serum (FBS) were obtained from GIBCO/BRL Life Technologies (Grand Island, NY). Sulforhodamine B (SRB), carboxyfluorescein succinimidyl ester (CFSE), propidium iodide (PI), acridine orange and all other chemical compounds were obtained from Sigma-Aldrich (St. Louis, MO). Antibodies to PARP-1, Bcl-2, Bcl-xL, Bid, Mcl-1, survivin, cyclin D1, cyclin E, cyclin A, cyclin B1, Cdk4, Cdk2, p21, p27, caspase-3, caspase-9, c-Myc, α-tubulin, anti-mouse IgG and anti-rabbit IgG were obtained from Santa Cruz Biotechnology, Inc. (Santa Cruz, CA). The other antibodies were from Cell Signaling Technologies (Boston, MA).

### Chemical synthesis

KUD983 (4-(*tert*-butyl) 2-methyl (2*S*,4*S*,5*R*)-1-((2*S*,3*R*,5*R*)-1-acryloyl-2- (4-bromophenyl)-5-(methoxycarbonyl)pyrrolidine-3-carbonyl)-5-(4-bromophenyl)pyrrolidine-2,4-dicarboxylate) and KUD984 (4-(*tert*-butyl)2-methyl (2*R*,4*R*,5*S*)-1-((2*R*,3*S*,5*S*)-1-acryloyl-2-(4-bromophenyl)-5-(methoxycarbonyl)pyrrolidine-3-carbonyl)-5-(4-bromophenyl)pyrrolidine-2,4-dicarboxylate) (Figure 1A) were synthesized according to the procedure for the racemic form [22] starting from (+)-4-(*tert*-butyl) 2-methyl (2*S*,4*S*,5*R*)-5-(4-bromophenyl)pyrrolidine- 2,4-dicarboxylate [61] and (–)-4-(*tert*-Butyl) 2-methyl (2*R*,4*R*,5*S*)-5-(4-bromophenyl)pyrrolidine-2,4-dicarboxylate [61], respectively. NMR characteristics of both compounds correspond to the racemic form NMR characteristics [22]. Elemental analyses of both samples were within ± 0.3 % range of theoretical values.

4-(*tert*-Butyl) 2-methyl (2*S*,4*S*,5*R*)-1-((2*S*,3*R*,5*R*)-1-acryloyl-2-(4-bromophenyl) 5-(methoxycarbonyl)pyrrolidine-3-carbonyl)-5-(4-bromophenyl)pyrrolidine-2,4-dicarboxylate (KUD983). White crystals, m.p. 144–146°C, [α]_*D*21_ +84.6° (*c* 1.10, CH_2_Cl_2_) > 99% *ee*(HPLC, Chiralcel OD).

4-(*tert*-Butyl) 2-methyl (2*R*,4*R*,5*S*)-1-((2*R*,3*S*,5*S*)-1-acryloyl-2-(4-bromophenyl)-5-(methoxycarbonyl)pyrrolidine-3-carbonyl)-5-(4-bromophenyl)pyrrolidine-2,4-dicarboxylate (KUD984). White crystals, m.p. 142–144°C, [α]_*D*21_ − 83.4° (*c* 1.25, CH_2_Cl_2_) > 99% *ee* (HPLC, Chiralcel OD).

### Cell lines and cell culture

Cancer cell lines PC-3 and DU-145 were from American Type Culture Collection (Rockville, MD). Cells were cultured in RPMI-1640 medium with 10% FBS (v/v) and penicillin (100 units/ml)/streptomycin (100 μg/ml). Cultures were maintained in a humidified incubator at 37°C in 5% CO_2_/95% air.

### SRB assays

Cells were seeded in 96-well plates. After 24 h, cells were fixed with 10% trichloroacetic acid (TCA) representing cell population at time zero (T_0_). After additional incubation of 0.1% DMSO or the compound for 48 h, cells were fixed with 10% TCA and SRB at 0.4 % (w/v) in 1 % acetic acid was added to stain cells. Unbound SRB was washed out. SRB bound cells were solubilized with 10 mM Trizma base. Using the following absorbance (515 nm) measurements, such as time zero (T_0_), control growth (C), and cell growth in the presence of compound (Tx), the percentage growth inhibition was calculated as: [1- (Tx-T_0_)/(C-T_0_)] × 100%.

### Cell proliferation assay with CFSE labeling

CFSE was dissolved in DMSO to constitute a storage solution of 10 mM and was kept at -80^o^C until use. The cells were adjusted to a density of 10^6^ cells/ml and were treated with CFSE at a final concentration of 10 μM. After incubation at 37°C for 10 min, labeling was blocked by the addition of RPMI medium with 10% FCS. Tubes were placed in ice for 5 min and then washed. After centrifugation, the cells were seeded in RPMI medium with 10% FCS in the absence or presence of the compound for 24, 48 or 72 hr at 37^o^Cunder 5% CO_2_/95% air. The fluorescence intensity was determined by flow cytometric analysis. The cell proliferation was followed by monitoring decrease in label intensity in successive daughter cell generations [62]. The proliferation index and the cell populations of parent or different generations were calculated by Modfit LT Version 3.2 and WinList Version 5.0 software.

### Cell cycle synchronization

Synchronization of the cells was performed by thymidine block. Briefly, Cells were treated with 2 mM thymidine in medium/10% FCS for 24 hr. After washing cells with PBS, the block was released by the incubation of cells in fresh medium/10% FCS (indicated as time zero), and cells were harvested at the indicated times. The cell-cycle progression was detected by flow cytometric analysis.

### Flow cytometric analysis of PI staining

After treatment, the cells were harvested by trypsinization, fixed with 70 % (*v/v*) alcohol at 4°C for 30 min and washed with PBS. The cells were centrifuged and resuspended with 0.5 ml PI solution containing Triton X-100 (0.1%, *v/v*), RNase (100 μg/ml) and PI (80 μg/ml). DNA content was analyzed with the FACScan and CellQuest software (Becton Dickinson, Mountain View, CA).

### DNA fragmentation assay

The DNA fragmentation was determined using the Cell Death Detection ELISAplus kit (Roche, Mannheim, Germany). The assay was based on the quantitative *in vitro* determination of cytoplasmic histone-associated DNA fragments (mono- and oligonucleosomes) after induced cell death. After the treatment with KUD983, the cells were lysed and centrifuged, and the supernatant was used for the detection of nucleosomal DNA according to the manufacturer’s protocol.

### Western blotting

After treatment, the cells were harvested with trypsinization, centrifuged and lysed in 0.1 ml of lysis buffer containing 10 mM Tris-HCl (pH 7.4), 150 mM NaCl, 1mM EGTA, 1% Triton X-100, 1 mM PMSF, 10 μg/ml leupeptin, 10 μg/ml aprotinin, 50 mM NaF and 100 μM sodium orthovanadate. Total protein was quantified, mixed with sample buffer and boiled at 90°C for 5 min. Equal amount of protein (30 μg) was separated by electrophoresis in 8 or 12% SDS-PAGE, transferred to PVDF membranes and was detected with specific antibodies (1:1000 dilution). The immunoreactive proteins after incubation with appropriately labeled secondary antibody (1:3000 dilution) were detected with an enhanced chemiluminescence detection kit (Amersham, Buckinghamshire, UK).

### Confocal immunofluorescence microscopic examination

After treatment, the cells were fixed with 100 % methanol at −20^o^C for 5 min and were incubated in 1 % bovine serum albumin (BSA) containing 0.1% Triton X-100 at 37°C for 30 min. Cells were washed and stained with primary antibodies at 37°C for 1 h and GFP-LC3 and cleaved casp3 antibody (Alexa flour 647 conjugate) at 37°C for 40 min. Nuclear identification was performed by DAPI staining. The cells were analyzed by a confocal laser microscopic system (Leica TCS SP2).

### Flow cytometric analysis of autophagy

Cell autophagy was assessed by detecting acidic vesicular organelles (AVO) using acridine orange. The cells were removed from the plate and centrifuged for five minutes. The cells were stained with 1 mg/ml acridine orange for 20 min at room temperature and finally the cells were collected and resuspended in PBS. Stained cells were analyzed using a FACSCalibur flow cytometer and Cell Quest software (BD, USA).

### Transient transfection

For transfection, PC-3 cells were seeded into 60-mm tissue culture dishes with 30% confluence and grown for 24 h to about 50% confluence. Each dish was washed with serum-free Opti-MEM (Life Technologies), and 2 ml of the same medium was added. Aliquots containing myr-Akt expression vector (2 μg) or a control plasmid in serum-free Opti-MEM were transfected into cells using Lipofectamine 2000 (Invitrogen) following the manufacturer’s instructions. After the incubation for 6 h at 37°C, cells were washed with medium and incubated in 10% FBS-containing RPMI-1640 medium for 48 h. Then, the cells were treated with or without the compound for another 24 h. The Western blot analyses were performed.

### Data analysis

Data are presented as the mean ± SEM for the indicated number of separate experiments. Statistical analysis of data for multiple groups is performed with one-way analysis of variance. Student’s *t*-test is applied for comparison of two groups. *P*-values less than 0.05 are statistically considered significant.

## SUPPLEMENTARY MATERIALS FIGURES


