# Supplementary material for: Enantiomerically pure β-dipeptide derivative induces anticancer activity against human hormone-refractory prostate cancer through both PI3K/Akt-dependent and -independent pathways
Source: Oncotarget. 2017 May 20;8(57):96668–83. doi: 10.18632/oncotarget.18040 (PMC5722513; doi:10.18632/oncotarget.18040)
Supplement: Supplementary file 1 [file oncotarget-08-96668-s001.pdf]

Enantiomerically pure β-dipeptide derivative induces anticancer activity against human hormone-refractory prostate cancer through both PI3K/Akt-dependent and -independent pathways

Supplementary Materials

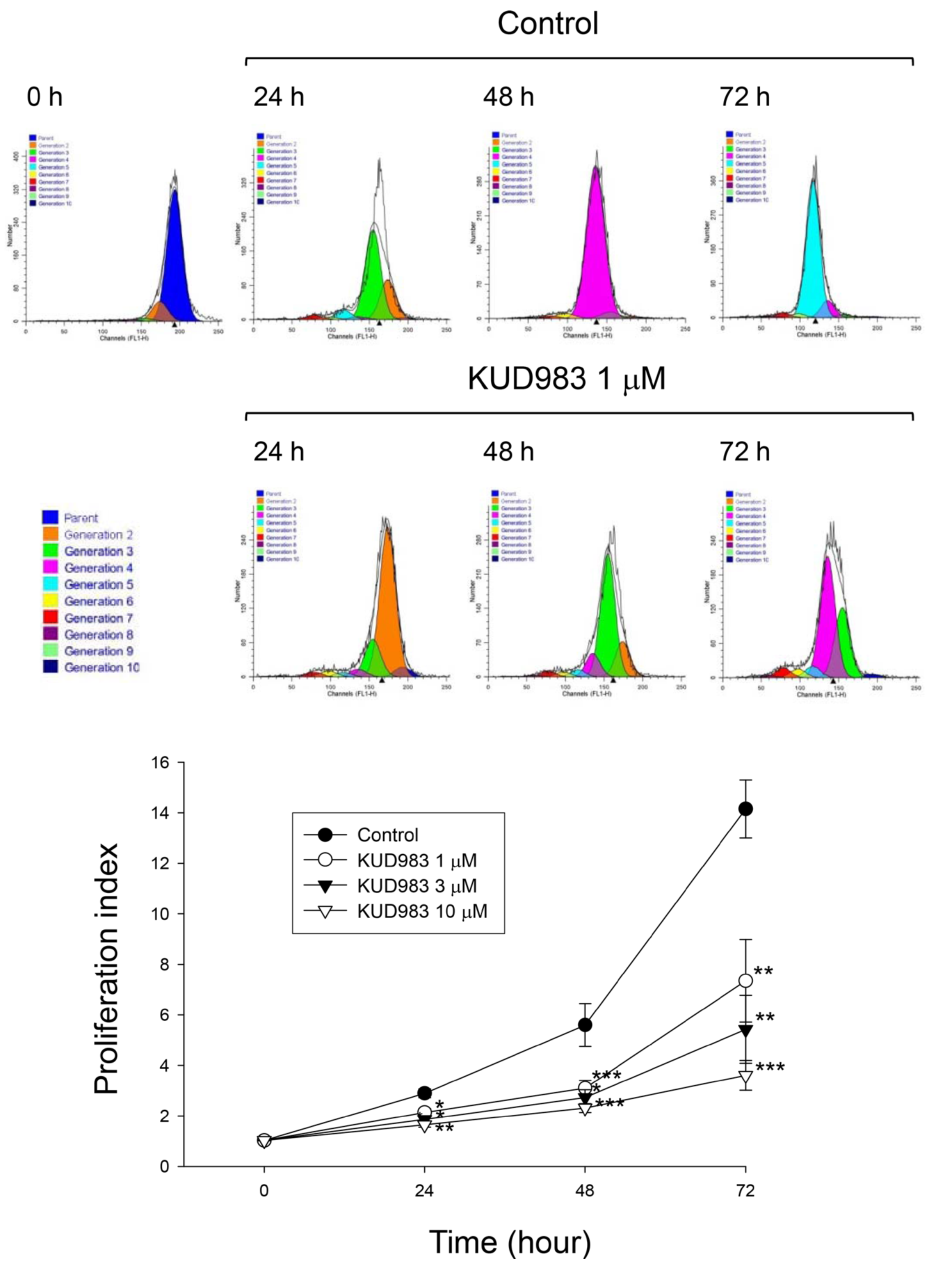

Supplementary Figure 1: Effect of KUD983 on cell proliferation in DU145 cells using CFSE staining assay. The indicated concentration of KUD983 was added to DU-145 cells for the 24, 48 or 72 h. After the treatment, the cells were labeled with CFSE for flow cytometric analysis. Gray curve, total cell counts; black curve, total cells in all generations; color area, population of different generation. Data are expressed as mean ± SEM of three determinations. $^{*}P < 0.05$, $^{**}P < 0.01$ and $^{***}P < 0.001$ compared with the respective control.

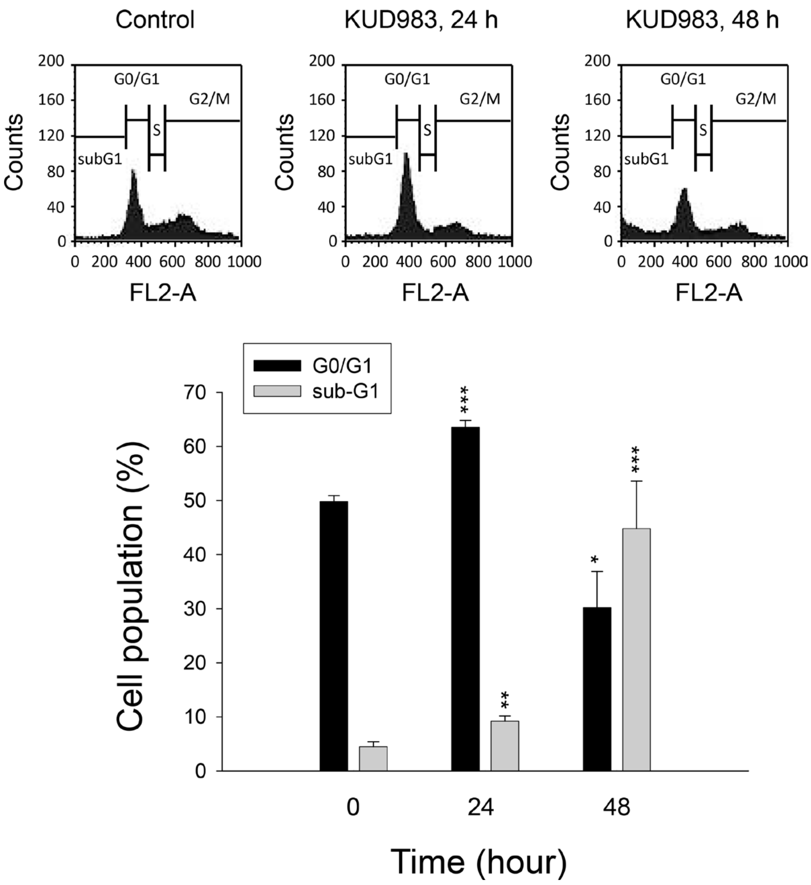

Supplementary Figure 2: Effect of KUD983 on cell-cycle progression. (A) DU145 cells were incubated in the absence or presence of KUD983 (3 μM) for 24 or 48 h. The cells were fixed and stained with propidium iodide to analyze DNA content by flow cytometric analysis. Data are expressed as mean ± SEM of three determinations. $^{*}P < 0.05$, $^{**}P < 0.01$ and $^{***}P < 0.001$ compared with the respective control.

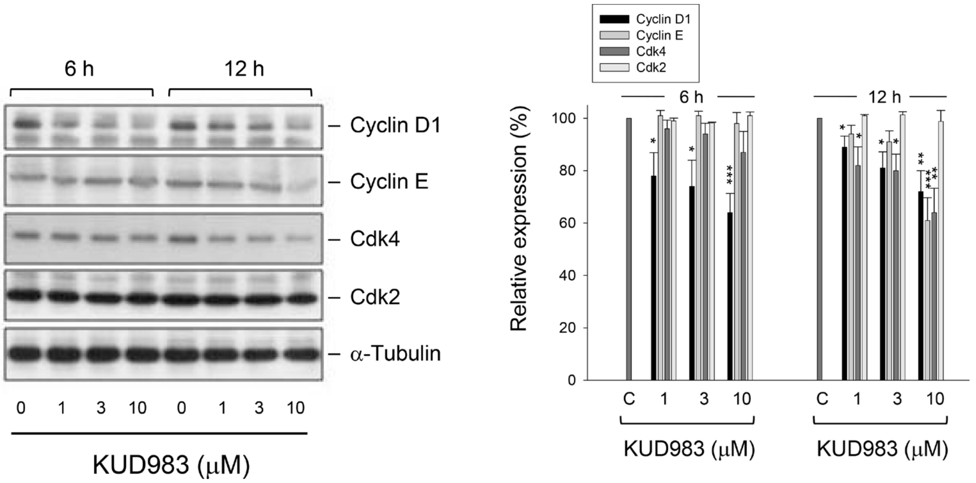

Supplementary Figure 3 : Effect of KUD983 on the expression of several cell cycle regulators. DU145 cells were incubated in the absence or presence of KUD983 for the indicated time and concentration. Cells were harvested and lysed for the detection of the indicated protein expression by Western blot analysis. The expression was quantified using the computerized image analysis system ImageQuant (Amersham Biosciences). The data are expressed as mean ± SEM of three to five independent experiments. $^{*}P < 0.05$, $^{**}P < 0.01$ and $^{***}P < 0.001$ compared with the control.

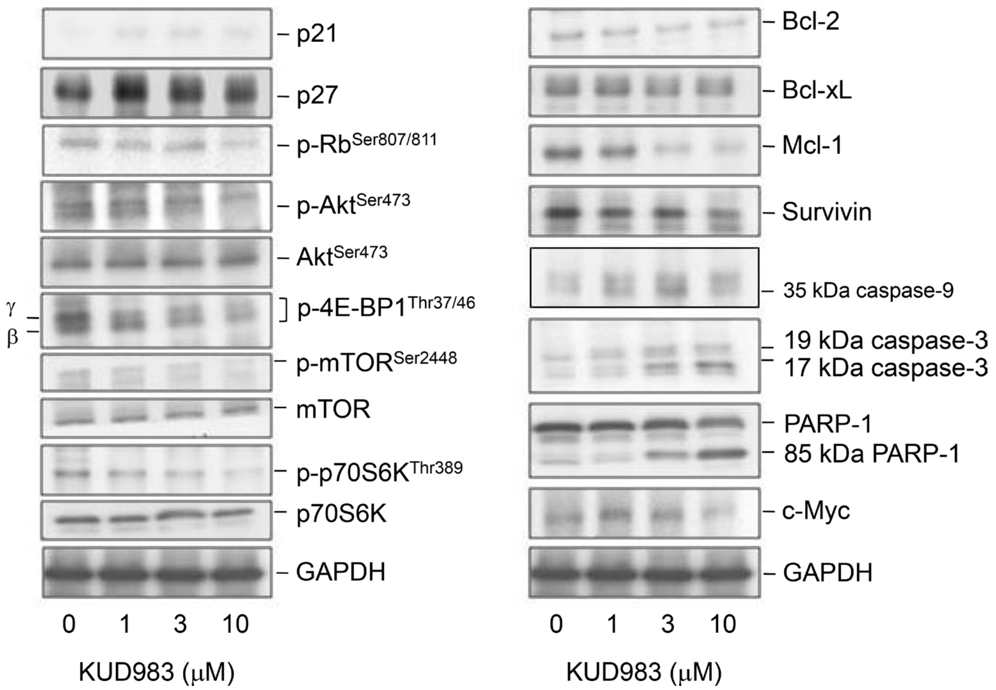

Supplementary Figure 4: Effect of KUD983 on the expression of several proteins. DU145 cells were incubated in the absence or presence of KUD983 for the indicated concentrations for 24 h. Cells were harvested and lysed for the detection of the indicated protein expression by Western blot analysis.

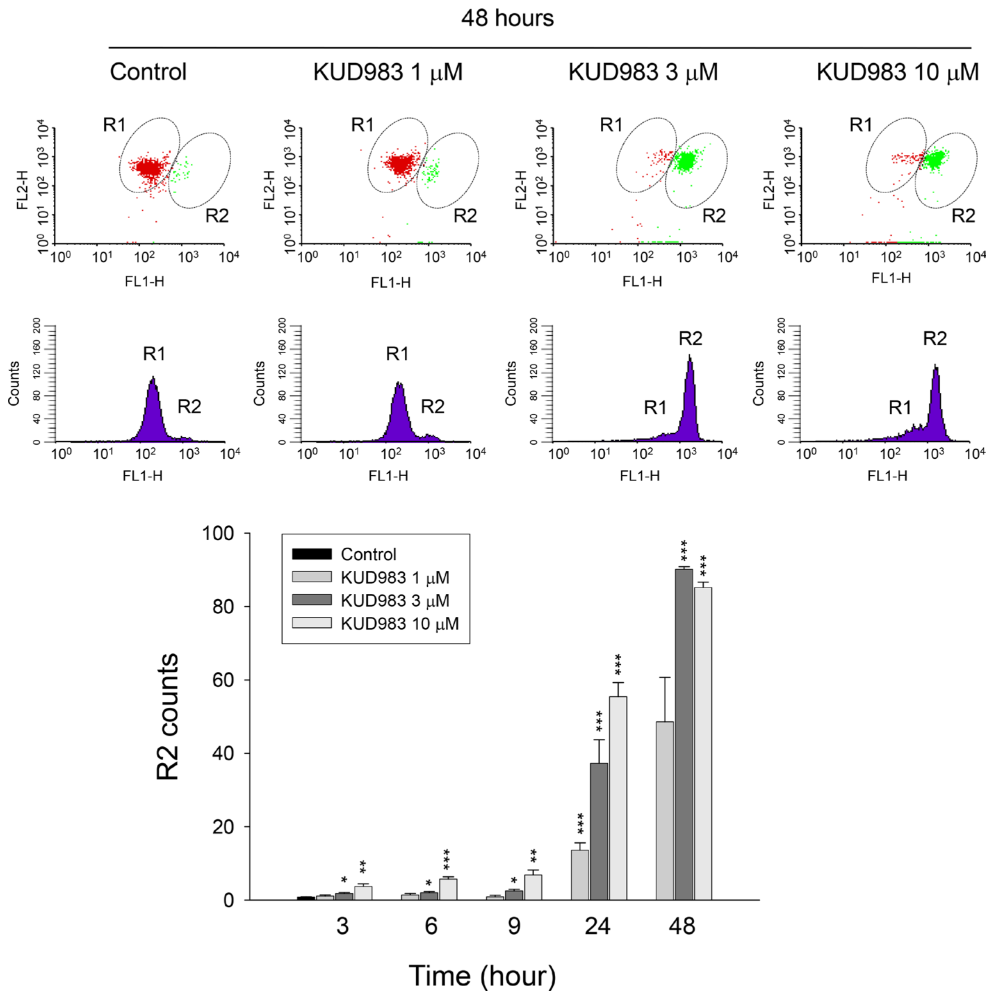

Supplementary Figure 5: Effect of KUD983 on mitochondrial membrane potential. PC-3 cells were treated without or with KUD983 for the indicated times. Cells were incubated with JC-1 for the detection of mitochondrial membrane potential using FACScan flow cytometric analysis. JC-1 aggregates (R1, red fluorescence) prefer higher mitochondrial membrane potential in cells under normal condition. After the loss of mitochondrial membrane potential, the JC-1 monomers are dominant with green fluorescence (R2). The data are expressed as mean ± SEM of four independent experiments. $^{*}P < 0.05$, $^{**}P < 0.01$ and $^{***}P < 0.001$ compared with the control.

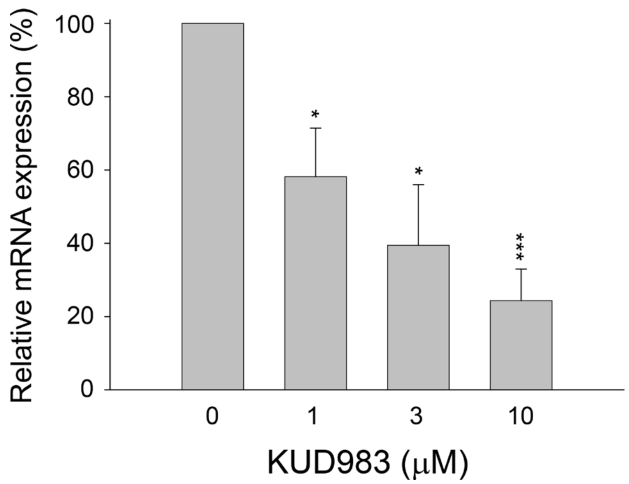

Supplementary Figure 6: Effect of KUD983 on cyclin D1 mRNA expression of PC-3 cells. The cells were incubated in the absence or presence of KUD983 for 6 h. The cells were harvested and the cyclin D1 mRNA levels were measured using real time PCR technique. The data are expressed as mean ± SEM of three independent experiments. $^{*}P < 0.05$ and $^{***}P < 0.001$ compared with the control.
